# Analysis of global nutrient gaps and their potential to be closed through redistribution and increased supply

**DOI:** 10.3389/fnut.2024.1396549

**Published:** 2024-08-09

**Authors:** Andrew J. Fletcher, Raquel Lozano, Warren C. McNabb

**Affiliations:** ^1^Riddet Institute, Massey University, Palmerston North, New Zealand; ^2^Sustainable Nutrition Initiative, Riddet Institute, Massey University, Palmerston North, New Zealand; ^3^Fonterra Research and Development Centre, Palmerston North, New Zealand

**Keywords:** systems modeling, micronutrients, sustainability, nutrient adequacy, mathematical modeling, inequality

## Abstract

Global food systems are crucial for sustaining life on Earth. Although estimates suggest that the current production system can provide enough food and nutrients for everyone, equitable distribution remains challenging. Understanding global nutrient distribution is vital for addressing disparities and creating effective solutions for the present and future. This study analyzes global nutrient supply changes to address inadequacies in certain populations using the existing DELTA Model^®^, which uses aggregates of global food production to estimate nutrient adequacy. By examining the 2020 global food commodity and nutrient distribution, we project future food production in 2050 needs to ensure global adequate nutrition. Our findings reveal that while some nutrients appear to be adequately supplied on a global scale, many countries face national insufficiencies (% supply below the population reference intake) in essential vitamins and minerals, such as vitamins A, B12, B2, potassium, and iron. Closing these gaps will require significant increases in nutrient supply. For example, despite global protein supply surpassing basic needs for the 2050 population, significant shortages persist in many countries due to distribution variations. A 1% increase in global protein supply, specifically targeting countries with insufficiencies, could address the observed 2020 gaps. However, without consumption pattern changes, a 26% increase in global protein production is required by 2050 due to population growth. In this study, a methodology was developed, applying multi-decade linear convergence to sufficiency values at the country level. This approach facilitates a more realistic assessment of future needs within global food system models, such as the DELTA Model^®^, transitioning from idealized production scenarios to realistic projections. In summary, our study emphasizes understanding global nutrient distribution and adjusting minimum global nutrient supply targets to tackle country-level inequality. Incorporating these insights into global food balance models can improve projections and guide policy decisions for sustainable, healthy diets worldwide.

## Introduction

The global food system is the most critical human activity, essential for sustaining the lives of everyone on the planet by providing the necessary nutrition (1). It also serves as a major economic activity and is responsible for a significant portion of the anthropogenic impact on the environment (2).

The ability of a country to secure food and nutrients for its population depends on factors such as agricultural production, trade dynamics, and import economic capacity (2, 3). This is impacted by international trade patterns, regional and national economic conditions, domestic food production and the resilience of food systems to external shocks from climatic events or political issues (3).

Previous study (4–6) aligning food production with the nutrient requirements of the global population has shown that with equitable global distribution, there was sufficient food produced in 2018 to meet nutrient requirements for everyone for 27 of 29 nutrients considered within the DELTA Model®. Projecting into the future, 2018 production included sufficient protein—and indispensable amino acids—to meet the requirements of the expected 2050 population if these were equally distributed (5).

These global-scale approaches assume equal access to nutrients for everyone on the planet and lead to the development of scenarios describing the minimum food production necessary to meet nutrient needs. This is itself a valuable insight as it gives us the minimum conditions under which it might be possible to adequately nourish everyone on the planet. However, the distribution of food and nutrients is not equitable, and with a level of global food production that could provide adequate nutrition for all, many are undersupplied. Developing scenarios for future food systems that accommodate some degree of inequality requires an understanding of how food commodities and nutrients are currently distributed.

The global food system is known for its complexity and wide-reaching impacts (2), illustrated by a variety of health outcomes. People may have protein-energy malnutrition, obesity due to excess energy consumption and lifestyle choices, and/or micronutrient deficiencies (often termed as ‘hidden hunger’). Micronutrient deficiency and obesity can exist at the same time as ‘hidden hunger’ or exist separately. To add further complexity, these issues can exist in the same country and the same household as those who do not have these health issues (7). Globally, in 2021, 768 million people were affected by hunger, 3.1 billion people were unable to afford a healthy diet, and simultaneously, 40% of all adults were overweight or obese (8). Dietary choices, availability, and affordability of food within a country play a significant role in these health outcomes and adequate nutrition through food and lifestyle choices is an effective strategy for avoiding long-term health consequences (9, 10).

By 2050, global populations will increase, and demographics will shift, leading to changes in global nutrient requirements. National food supplies are expected to encounter pressures due to the impact of climate change on domestic food production, impacting the ability to consistently meet market demands and uphold nutritional requirements (4, 11).

Bell et al. (12) investigated several aspects of inequality in global food, nutrition, and health between 1970 and 2010. These included energy intake from animal-sourced foods, energy intake from fruits and vegetables, intake of vitamin A, zinc, and iron, and health indicators like child stunting and the prevalence of overweight and obesity in men and women. Their study determined and compared global distributions of these nutrients and health metrics at both ends of this period, while also considering factors such as food production, land use, and GDP *per capita*.

In our study, we seek to understand current (2020) inequality in nutrient supply and use this to consider the impact on future food system scenarios with a view to better-informing conversations about how the food system might change to deliver sustainable development goal #2 of Zero Hunger. Understanding the present state of global nutrient distribution is a crucial step in identifying the areas of disparity and creating credible solutions. Food supply is linked to nutrient distribution and is a key component of both sustainable food systems and sustainable healthy diets.

Initially, the study modeled the current distribution of nutrient supply at the country level against population requirements. This process generated both a global sufficiency distribution for each nutrient and country-specific sufficiency patterns across all the nutrients. Subsequently, the research aimed to utilize this information to address the following questions:

1. In a scenario where the total global nutrient supply is sufficient, what adjustments are necessary to close nutrient inadequacies by redistribution from those who have more than enough? What approaches, or foods, could help countries with shortages to secure adequate nutrient supply?
2. Looking into the future, what is the impact of unequal distribution of food and nutrients on the food production required to deliver sufficient nutrition to everyone on the planet? How much more of each nutrient is required to accommodate the reality that many people consume more than the minimum requirements for health, thus potentially depriving others of an adequate intake?

This study provides a method to set revised minimum supply targets for future scenarios in a manner that accommodates inequality at a country level and to apply these in global food nutrient balance models such as the DELTA Model^®^ (5) to deliver more realistic projections for the future. For example, an oft-quoted statement is that we need to increase global protein production by 70% by 2050 to meet the “needs” of the changing population (13), yet the study by Smith et al. (5) shows we could make do with current production. Which is correct? This study provides another approach to answering the future supply question for protein and other nutrients.

## Methods

### Quantifying nutrient gaps

Data from the DELTA Model^®^ (version 2.2) were used for this analysis. The methodology used to calculate the nutrient supply at a country level is detailed in a previous study by Smith et al. (5) but is described briefly here. In this study, global supply values from 2020 were used.

The DELTA Model^®^ used the food balance sheets from the United Nations Food and Agriculture Organization (14), which contains the total supply of food items intended for human consumption after trade, non-food uses, and supply chain losses. Food item quantities are further adjusted for consumer waste using a second FAO source (15).

The food items are matched to food composition data from the United States Department of Agriculture to calculate the total quantity of nutrients on a country basis. For protein and the indispensable amino acids (IAAs), the values are adjusted for digestibility using true ileal digestibility coefficients from literature sources (16, 17). The results can be compared to national nutrient requirements, which are calculated using demographic data for the age and sex proportions of the population and the European Food Safety Authority (EFSA) nutrient reference values (16, 17). The target intake values are defined as the population reference intakes (PRI, where available) or adequate intakes when PRI is not available. IAA requirements are determined from the protein requirements (g/kg body weight) and the reference amino acid patterns (g/kg protein). The method is the same as used in the DELTA^®^ model, and the corresponding section of the supplementary material from (5) is included in the Supplementary material.

**Figure 1:**
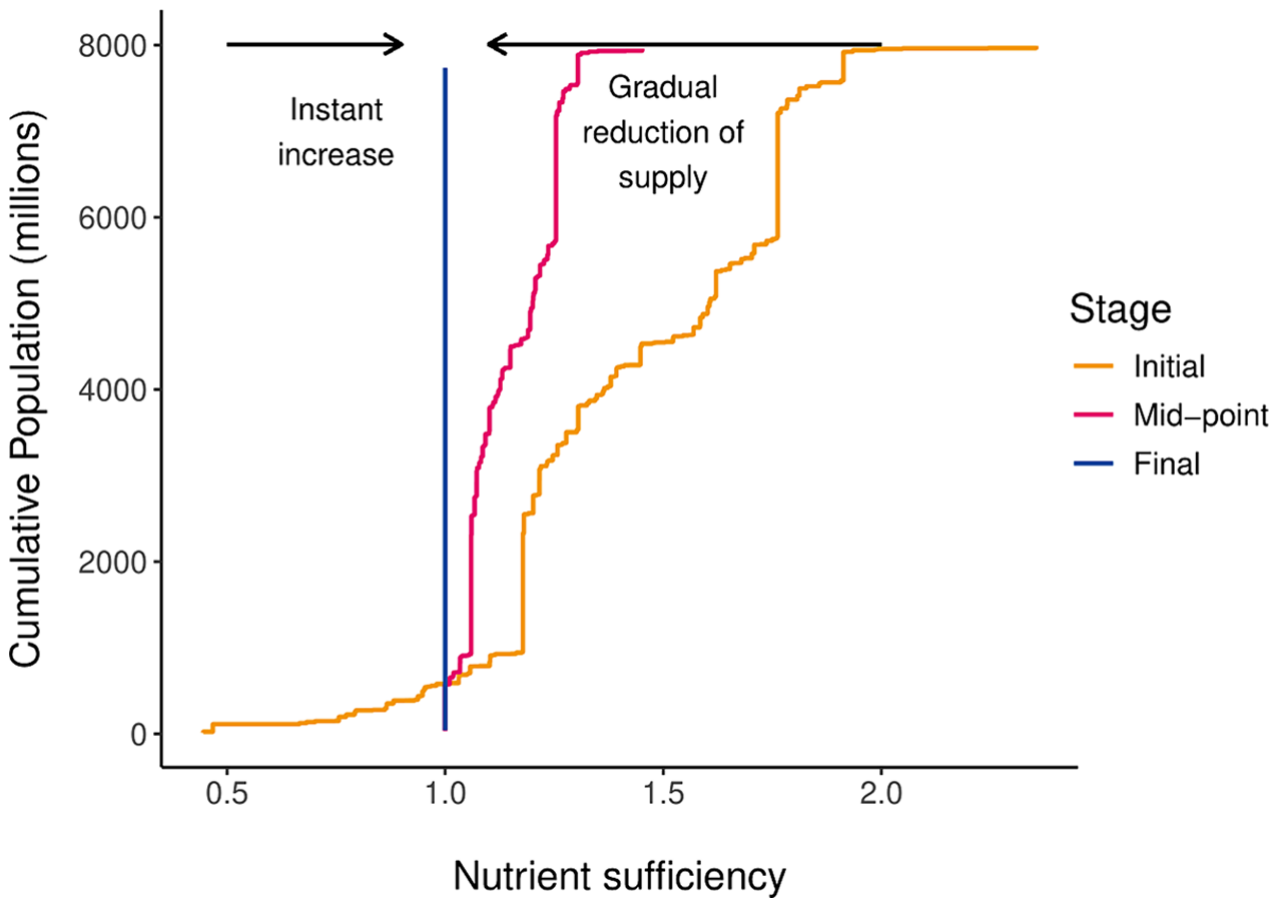
Conceptual diagram of nutrient distribution and re-distribution across the global population. Colors and linetype represent the stage of supply reduction and text labels, and arrows indicate the direction of increase or decrease.

The ratio between the current supply of a country of a given nutrient and its target intake determines the sufficiency ratio. This sufficiency ratio is used as an indicator to display whether current nutrient supplies are adequately meeting national target intake values. Figure 1 is introduced here to illustrate the method. The set of national sufficiency ratios provides a sufficiency distribution across the global population as illustrated by the solid “Initial” line. This does not consider the impact of within-country variation in food intake and nutrient supply, which adds additional variation. Country-level nutrient sufficiency is a necessary—but not sufficient—condition for nutrient adequacy within a population.

In a utopian scenario, with equitable global distribution and consumption, the sufficiency ratio of all countries for a given nutrient is equal. Global sufficiency is achieved by ensuring the average global supply exceeds the average global requirement. This is the default use of the DELTA Model^®^, which leads to the design of bare minimum scenarios for global food and nutrient supply scenarios, in which it is “possible” to meet global nutrient requirements.

The variation that exists between countries means that even when average global sufficiency is well above 1.0 there may be a significant proportion of the global population that live in countries that have an insufficient supply. For the years covered by the food balance sheet data—in this case 2020—the global nutrient gap can be calculated as the additional amount of a nutrient required to have brought all countries with insufficient supply up to a sufficiency value of 1.0 without changing the supply to the other countries. This is expressed as a percentage increase in the global supply of the nutrient.

### Global scenarios for nutrient adequacy

When creating food production scenarios for future years, the challenge is setting realistic and practical nutrient supply targets that have the potential to ensure that all global citizens have access to an adequate supply. The first aspect of this is straightforward in setting the minimum sufficiency target for nations with an inadequate supply to 1.0.

The second aspect is setting expected future sufficiency levels for countries currently enjoying a more than adequate supply. Consumption of most nutrients above the required level does not cause harm to the individual, and people derive considerable pleasure from eating food. However, at high intake levels, some nutrients may be toxic (18). Apart from reducing energy intake the nutritional benefit of an individual reducing intake of a nutrient that is currently oversupplied is abstract and remote—more nutrients available for someone else—which limits the drive for rapid change, unless driven by external forces (availability, affordability). Even when individual change occurs rapidly, an extension of change over groups, countries, and globally takes much longer and changes are complex to implement due to the inherent interconnected nature of food systems (2). A reduction in nutrient intake by those currently enjoying a surplus should realistically be seen as a decades-long process and, to be “practical,” future scenarios should recognize this. Changing systems at a gradual rate, as one of the options presented in this study, would present less long-term stress on the food system while allowing for the necessary changes.

One approach is to set the nutrient “needs” of countries currently enjoying more than sufficient supply to linearly reduce from current levels to converge with the basic requirement in a future year (e.g., 2050). Combining these two aspects enables future minimum sufficiency targets for countries to be set by Eq. 1.


(1) $$S_{i,j}\left(k\right)=1+\max\;\left(\frac{y-k}{y-2020}\left(S_{i,j}\left(2020\right)-1\right),0\right)$$

where S is the sufficiency ratio, i is the nutrient, j is the country, k is the year of interest, and y is the convergence year (e.g., if *S*(2020) = 1.6, *y* = 2050 and *k* = 2040 then *S*(*k*) = 1.2).

A slightly modified approach that allows setting a sufficiency convergence point $S_{i}\left(y\right)$ that is greater than 1.0 is given by Eq. 2.


(2) $$S_{i,j}\left(k\right)=S_{i}\left(y\right)+\frac{y-k}{y-2020}\left(\max\left({S_{i,j}}^{*}\left(2020\right),1\right)-S_{i}\left(y\right)\right)$$

Summing across the globe, we can calculate the required global nutrient sufficiency to achieve the specified transition path. This involves summing up the required alterations in supply for each country.

## Results

### Quantifying nutrient gaps

**Figure 2:**
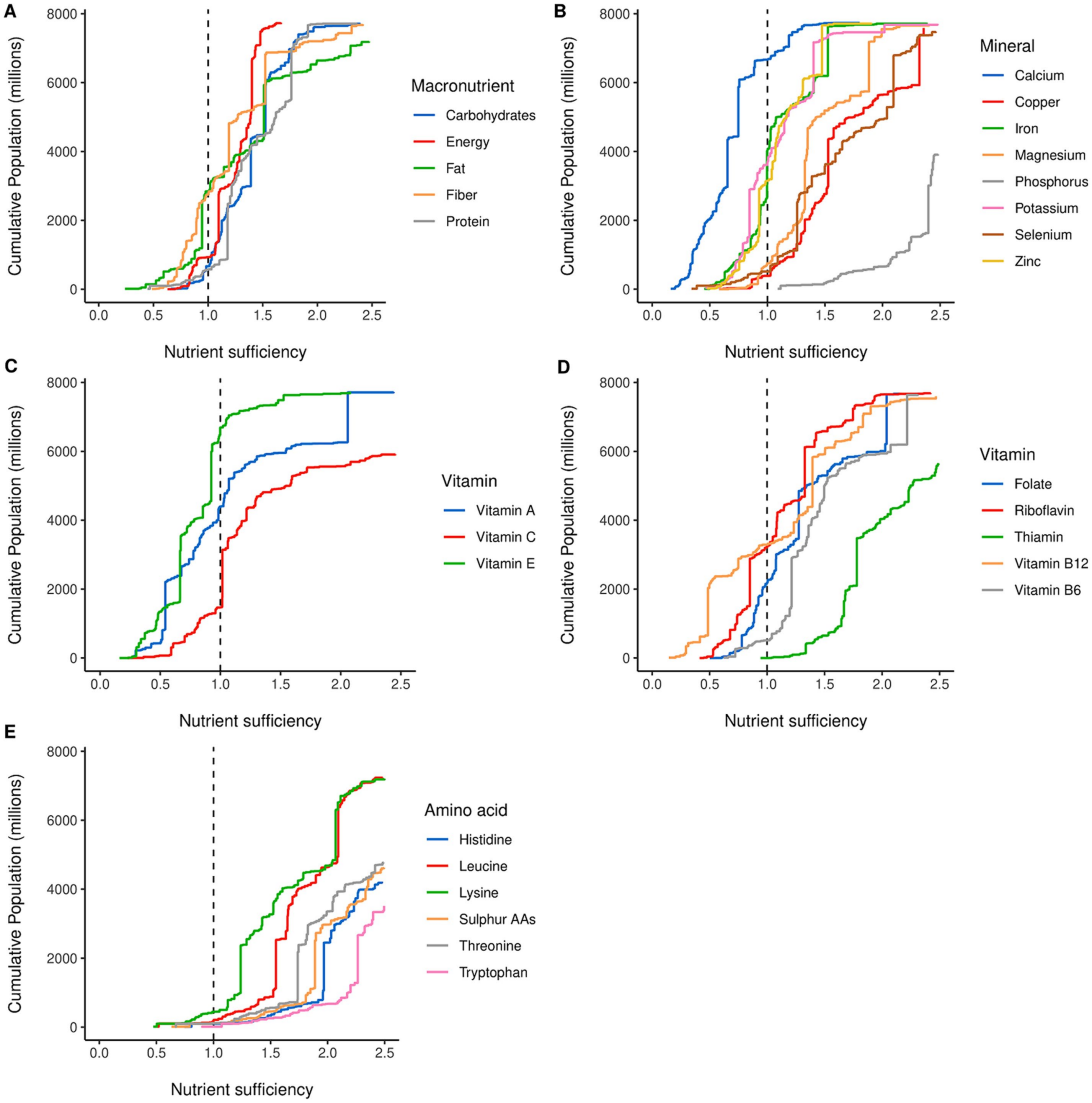
Cumulative distribution of nutrients across the global population in 2020. The *x*-axis denotes the nutrient sufficiency at a country level; the *y*-axis is the global cumulative population. **(A)** Distribution for macronutrients, **(B)** distribution for minerals, **(C,D)** distribution for vitamins, separated between non-B and B vitamins, and **(E)** distribution for indispensable amino acids. Colors are individual nutrients indicated by the subplot legend.

The distribution of nutrients for the global population is displayed in Figures 2A–E. Results show a range of nutrient disparities across the global population.

The macronutrient results (Figure 2A) show considerably narrower ranges of nutrient sufficiency compared to other nutrient groups such as the amino acids, seen in Figure 2E. This is not that surprising as macronutrients are linked to food bulk and satiety, limiting consumption at the upper end, and shortages (particularly of energy and protein) have acute and severe consequences at the lower end. Fat was the most limiting macronutrient in this analysis with 2.84 billion people in countries with an inadequate supply although approximately 1.7 billion of these are within 10% of the target level. Protein was the least limiting macronutrient with 570.3 million people short, and fiber showed the largest range among the macronutrient results with some groups having a supply 3.5 times greater than the target intake value.

Figure 2B shows that calcium is the most limiting nutrient overall with approximately 6.7 billion people in countries that appear to have insufficient dietary calcium supply based on calcium derived from primary food products and without considering fortification or supplements. This contrasts with phosphorous where the global population is well supplied. The remainder of the minerals studied form two distinct groups: iron, zinc, and potassium show very similar distribution curves with approximately half of the global population at or below an adequate supply; copper, magnesium, and selenium form another cluster with much lower national deficits.

The vitamins were split into two groups to increase visibility as can be seen in Figures 2C,D. Tail of vitamin B12 is the result of Mongolia and Hong Kong having sufficiency ratios of 8.4 and 9.2, respectively, which stems from the large amount of meat (including offal) reported as available as food in these countries. Vitamin B12 had the lowest minimum, and highest maximum, nutrient sufficiency ratios of all the nutrients considered in this study. Vitamin E had similar results to calcium, with approximately 6.7 billion people in countries currently undersupplied with reference to the target intake. Thiamin had one of the lowest nutrient gaps with approximately 210.5 thousand people affected by nutrient insufficiency worldwide (0.003% of the global population).

The IAAs show similarly shaped distributions. This group is the least limiting compared to the other nutrient groups, with most countries having an adequate supply for almost all the IAAs. Lysine was the most limiting IAA with 443 million people (5.7%) in countries currently undersupplied. When comparing lysine and overall protein sufficiency ratios, all countries with sufficient protein also had sufficient lysine, except for Afghanistan where the lysine sufficiency ratio was 0.8.

Table 1 shows the summary results for 2020 and includes a calculation of the minimum increase required to close the nutrient gap for all countries with insufficient supply without reducing the supply for those who were above a sufficiency ratio of 1.0. For the IAAs, only small increases in supply are needed, as most of the population is well supplied and the high global sufficiency levels indicate that by redistribution it could be possible to decrease supply and yet maintain the entire global population above 1.0. The minimum increases in supply were less than 10% for the other nutrients, except for calcium (51%), vitamin E (30.9%), vitamin A (17.8%), and vitamin B12 (16.3%).

**Table 1:** Summary results by nutrient showing global sufficiency value in 2020, the proportion of global population living in countries without a sufficient supply, the minimum increase in global supply required to bring all countries to basic sufficiency, and top 10 rankings based on the sufficiency score and minimum change.

Nutrient	Global sufficiency	Top 10 ranking by global sufficiency	Population in countries undersupplied as %	Minimum change required as %	Top 10 ranking by minimum change
Macronutrients					
Energy	124%	10	12.1%	1.5%	
Carbohydrate	139%		8.6%	0.5%	
Fat	137%		36.8%	4.1%	10
Fiber	122%	9	35.5%	5.4%	8
Protein*	143%		7.4%	1%	
Amino acids*					
Histidine	244%		1.3%	0.1%	
Leucine	183%		2.1%	0.3%	
Lysine	171%		5.7%	0.7%	
SAA (Cys + Meth)	233%		1.5%	0.1%	
Threonine	222%		1.5%	0.2%	
Tryptophan	272%		0.2%	~0%	
Minerals					
Calcium	68%	1	86.1%	51%	1
Copper	168%		5%	0.4%	
Iron	110%	6	51.9%	6%	7
Magnesium	145%		9.7%	0.5%	
Phosphorous	268%		0%	0%	
Potassium	109%	4	46.6%	8.2%	5
Selenium	166%		6.7%	1.1%	
Zinc	110%	5	40.6%	5.4%	9
Vitamins					
A	108%	3	56.8%	17.8%	3
B1—Thiamine	212%		0%	0%	
B2—Riboflavin	114%	7	42.1%	8%	6
B6—Pyridoxine	152%		6.6%	0.9%	
B9—Folate	135%		28.3%	3%	
B12—Cobalamins	115%	8	42.5%	16.3%	4
C	154%		19%	3%	
E	80%	2	86.5%	30.9%	2

^*^Protein and amino acid sufficiency values are given after adjusting for ileal digestibility.

Comparing the percentage of the global population undersupplied and the change in supply required to address this provides some interesting results. Iron, for example, showed that while 51.9% of the population did not have adequate supply, a 6% increase in supply would be sufficient to close the gap as many countries were very close to an adequate supply. In comparison, vitamin B12 showed a lower value for the proportion of the population undersupplied and a higher overall global sufficiency than iron; however, as there was a much wider distribution between countries, a much larger increase (+16.3%) was required to close the undersupply gap. These results demonstrate the importance of examining the inter-country distribution of nutrients as well as global adequacy when considering the performance of the current or a proposed future food system. Upon ranking based on global sufficiency and minimum change criteria, the order of priority shifted. Specifically, vitamin B12 ascended from eighth to fourth place, while zinc descended from fifth to ninth.

**Figure 3:**
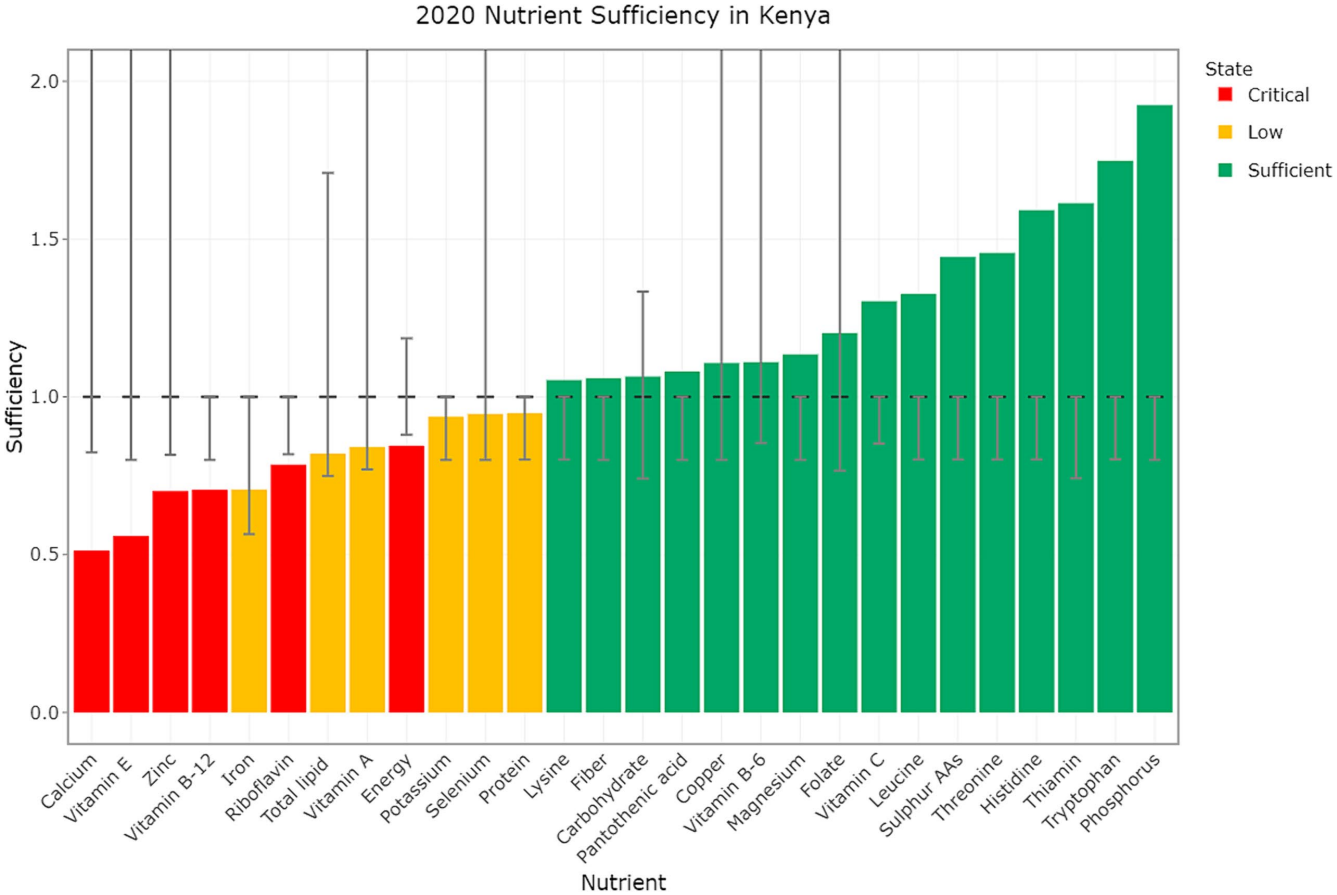
Nutrient sufficiency in Kenya for the Year 2020. The bars represent the level of sufficiency, while the dashed black line indicates the threshold for nutrient sufficiency based on PRI. The lower limit is either the EAR if this is available or 20% below the target if not. Error bars denote upper and lower limits where applicable, and colors signify different states of sufficiency with values below the lower limit shown as critical (red), values between the lower limit and the target as low (gold), and values at or above the target as sufficient (green). Upper limits are only shown where these fit within the *y*-axis bounds.

The same data can be used to look across the supply of all nutrients for a single country. For example, Figure 3 shows the 2020 nutrient sufficiency estimates for Kenya. These show supply gaps for five minerals and four vitamins in addition to protein and total calories. Targeting nutrient adequacy here requires foods that are good sources of calcium, vitamin E, zinc, vitamin B12, etc. The equivalent charts for the other countries covered by the FAO Food Balance Sheets are available within the latest version of the DELTA Model^®^.

### Global scenarios for nutrient adequacy

**Figure 4:**
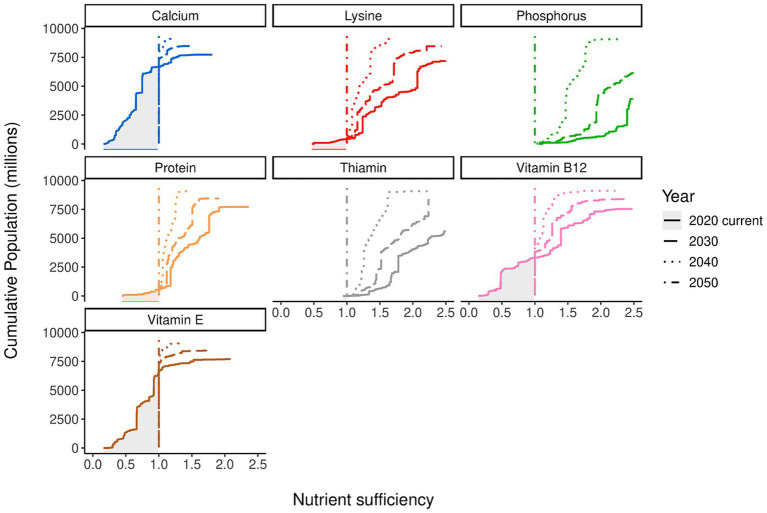
Potential change in country-level nutrient sufficiency based on a future convergence date of 2050. Colors indicate nutrients, with the linetypes indicating the year, which includes the population demographics and prospects in that year. The *y*-axis is the cumulative population; the *x*-axis is the nutrient sufficiency ratio.

Figure 4 shows the possible changes for a selection of nutrients when insufficiencies are resolved in countries that have them and oversupplied countries have their supply reduced to 1 over a 30-year convergence period (from 2020 to 2050). Results for all other nutrient groups can be found in the Supplementary material. A comparison of the curves shows the impacts of the linear reduction model application. For calcium and vitamin E, much of the population was undersupplied, and filling the shaded area dominates future changes. This contrasts with the protein, phosphorous, lysine, and thiamin where the changes are dominated by a reduction in supply above the sufficiency line.

**Figure 5:**
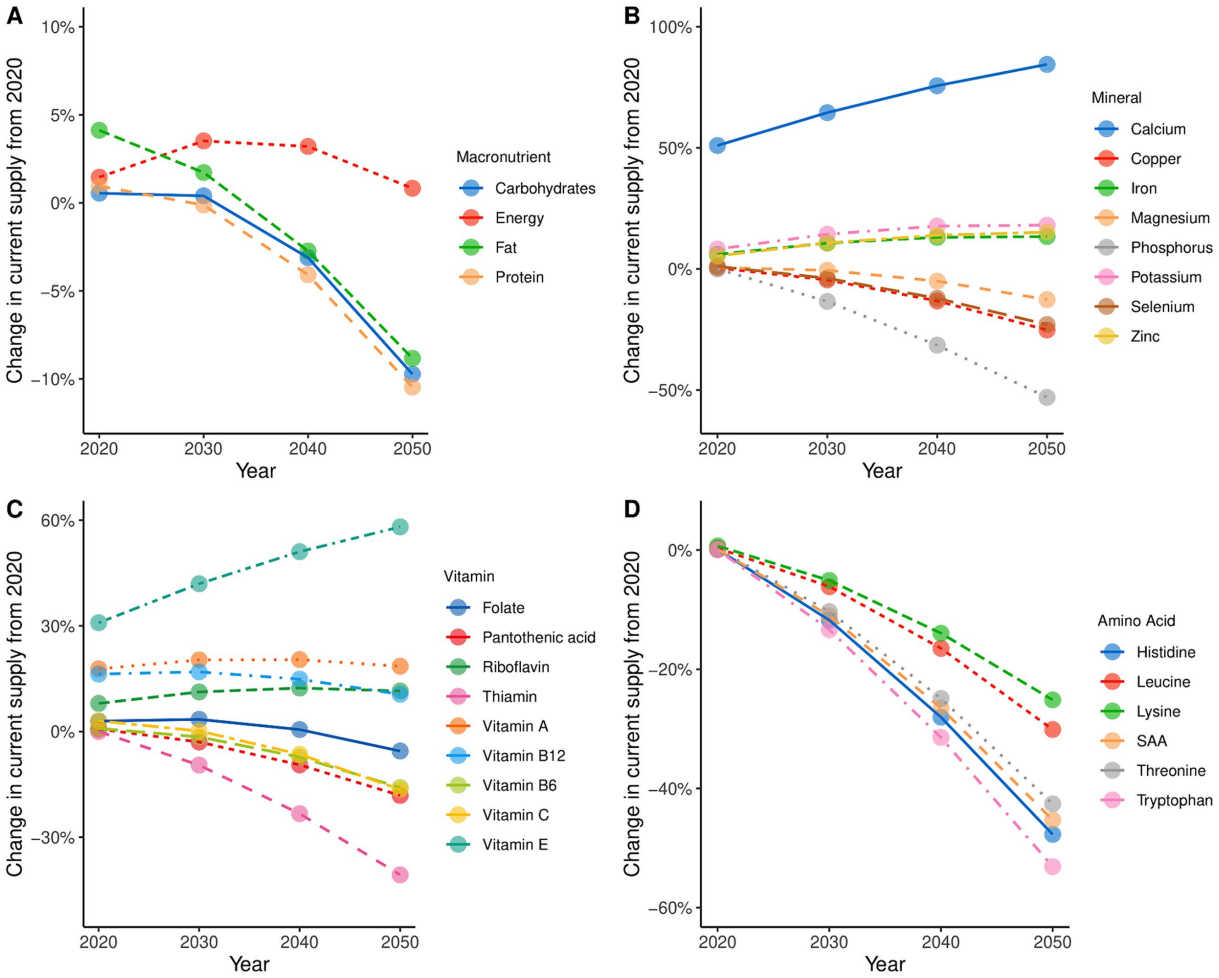
Required changes to global nutrient supply compared with 2020 in order to meet the minimum requirements for all countries. **(A)** shows the changes in macronutrients, **(B)** shows the changes in minerals, **(C)** shows the changes in vitamins, and **(D)** shows the changes in amino acids. The *y*-axis shows the percentage change with reference to the current 2020 supply and is scaled to the nutrient group. The *x*-axis is the year. New supply values are based on the change in the target intakes for the future population. The new target intakes are based on the linear reductions from the oversupplied parts of the population and the increase for those who are undersupplied based on a convergence date of 2050. The *y*-axis scales vary significantly between the nutrient groups.

The effects of redistributing nutrients from all countries, as depicted in Figure 3 (with Kenya as an example), on total nutrient requirements can be observed in Figures 5A–D. This is shown as a percentage change in global supply over 2020–2050 required to deliver the minimum nutrient requirements of the 30-year transition model. The starting point for all the curves is the 2020 supply that would have been required to meet minimum requirements for all countries without decreasing supply where this was more than adequate.

The curves show interesting differences. For some nutrients, supply must increase between 2020 and 2030 as the nutritional demands of changing global demographics exceed the amount released by reductions elsewhere. For calcium (Figure 5B) and vitamin E (Figure 5C), a decrease in minimum supply is never achieved. A significant shift in the current supply of calcium and vitamin E is required to achieve nutrient sufficiency. Energy and fiber show a peak between 2030 and 2040 before decreasing to a final supply near the 2020 starting point (after closing gaps). Minerals and vitamins in Figures 5B,C show a diverse set of changes where some nutrients show significant increases (calcium and vitamin E) and others such as phosphorus and thiamin show substantial decreases. The IAAs (Figure 5D) show a significant reduction as the redistribution effects allow less nutrients to be produced, despite increases in population compared to 2020. Another way of reading these curves is that when values decrease over time in this manner the nutrient is unlikely to be limiting for global nutrition.

## Discussion

### Intercountry comparison of nutrient distribution

Results showed the current distribution of nutrients (both macro and micro) across the global population. Our study shows that the world’s 2020 food supply could—with the exceptions of calcium and vitamin E—nourish the world’s population, but that unequal distribution of food means that for almost all nutrients there is a portion of the population that is not adequately supplied. Studies by Wood et al. (19) and Wang et al. (20) have come to similar conclusions. In the study by Wood et al. (19), the global food system, including food trade, waste, and conversion of food to non-food uses, was examined to highlight current trends and identify the nutritional potential of the food system. The authors found that there was sufficient food globally to meet global nutrient demands if equally distributed for the year 2018, with folate being the most limiting nutrient. However, Wood et al. (19) also suggest there is significantly more capacity in the food system, including the ability to meet the protein needs of an additional 11 billion people by redistribution of excess consumption, which does not align with our findings.

A more recent study by Wang et al. (20) found that calcium, vitamin B12, vitamin B6, iron, vitamin A, and zinc were undersupplied globally, which aligns with the trend in our results. However, they arrived at significantly lower figures for global food and nutrient supply, despite using similar data sources and methods. It appears that Wang et al. (20) subtracted pre-harvest or on-farm losses from the Food Balance Sheet production data, where it is our understanding that the FBS production figures represent the commodities that leave the farm or fishery (and thus enter the government production statistics used as the basis of the FBS) and have already accounted for such losses. Wang et al. (20) presented results depicting the sufficiency of nutrients across global populations, aggregated by regions, where we have taken a per-country approach.

A significant outcome of our study is highlighting the connection, and contrasts, between global nutrient sufficiency and the impact of food distribution. Take iron, for instance; the global supply is 110% of requirements, yet 51.9% of individuals are in countries that are undersupplied to varying degrees. Wang et al. (20) indicated that iron was almost sufficiently supplied but identified moderate deficits in Southeastern Central Asia, Oceania, Sub-Saharan Africa, and Latin America—consistent with the shortfall for iron observed in our results. The study at hand did not incorporate a regional analysis, contrasting with the approach of Wang et al. (20). However, future research endeavors could aim to identify regions experiencing nutrient insufficiency alongside high trade activity and elevated GDP *per capita*, similar to the study presented by Bell et al. (12). This endeavor could begin to create a perspective on the linkage between nutrient supply distribution and broader global system dynamics. Insights from the nutrient trade dynamics presented by Smith et al. (5) may offer valuable guidance for such prospective investigations.

A further layer of complexity exists when considering the variability within countries due to dietary choices, food availability, and affordability. To examine the intra-country nutrient distribution, Passarelli et al. (21) took a bottom-up approach and used dietary data sets from 31 countries to model nutrient adequacy against estimated average requirements (EAR) for a range of nutrients. This demonstrated significant within-country variation and significant differences between women and men, with women generally having less adequate intakes. Within-country intake distributions tend to be skewed with a tail toward the upper end, these distributions require at least three parameters (mean, coefficient of variation, and skewness) to be properly characterized, and the shape of the distribution has a significant impact on predictions of the portion of the population with inadequate intakes. Even where national-level supply appears to be adequate, a large portion of the population may have inadequate intakes (22). This within-country variation is additional to the between-country variation we have characterized.

Within this study, we have used PRIs as these represent the amount of nutrient required per person to meet the needs of 97.5% of the population within each of the gender and age bands. If a country has sufficient supply to meet the demographically weighted PRI, then in the absence of distribution inequality within the country this provides enough for the needs of almost every citizen. The alternative approach of using EARs would imply that for any country that just meets the target level for a nutrient, 50% of the population would be adequately supplied and 50% undersupplied, even without considering the impact of internal distribution effects.

Duro et al. (23) constructed a simplified food index to assess the resource use of food supply of different countries based on the portion of food energy from plant and animal sources, with the animal fraction multiplied by five to reflect the greater feed and thus cropland demand and presented this by country income category between 1990 and 2013. For high-income countries, this remained almost constant, low-income countries had a slight increase, and intermediate categories showed larger increases—in particular, China. They used the Theil index (a measure of inequality) which showed a decrease in inequality over the period of study—from 0.075 to 0.05. D’Odorico et al. (24) calculated Gini coefficients for food at a country level using a calorie-based analysis and showed that the level of inequality in food production (0.57 in 2010) was significantly greater than for food availability (0.23 in 2010). Our findings suggest that food availability remains a significant concern, and there is potential for increasing inequality if we persist on the current trajectory. D’Odorico et al. (24) also noted “Although the existence of country-average food availability above the malnourishment level is an important prerequisite for food security, within-country inequalities may still prevent part of the populace from having adequate access to food.” Future research in this field could explore nutrient distribution within countries to determine whether well-supplied nations are more likely to have adequate nutrient provision across their regions, even when the overall food supply meets nutrient sufficiency criteria. In a similar study Bell et al. (12) investigated inter-country distributions of agriculture production and health status metrics, and a range of nutrients between 1970 and 2010. To measure inequality, Gini coefficients were given for all variables in both 1970 and 2010 and largely show a reduction in inequality at a country level over this period. Our study starts with the level of nutrient inequality present in 2020 and generates global nutrient supply targets that would bring all countries to an equal and adequate supply in the chosen convergence year of 2050.

A challenge in changing nutrient intakes is that we consume foods, not nutrients, and changes need to be considered from the perspective of an individual’s food intake or the production of foods at regional, national, and global scales. The apparent oversupply of many nutrients is often the consequence of consuming foods that are critical to achieving sufficiency of less abundant nutrients, and large reductions are unlikely to be realized, unless the constrained nutrient is delivered from an alternate source. Translating this back into food production or dietary scenarios requires the use of tools such as the DELTA Model^®^ or dietary nutrient models that link nutrient supply to foods produced or eaten.

Limitations to these results include uncertainty on the final form in which the foods are consumed, which may impact the nutrient content, both from potential loss of nutrients through processing and food preparation, and not allowing for fortification of micronutrients where this is common practice. Beal et al. (22) showed that fortification has reduced micronutrient deficiencies in many developed countries; however, many low-income countries—where the need is greatest—do not have fortification legislation in place. Selection and development of crop varieties with high levels of micronutrients (biofortification) is also an option that is not currently considered in the modeling. Another limitation of this study is the variability in food composition data, particularly for minimally processed foods (25–27). Addressing this variability could involve using location-specific food databases, instead of assuming global consistency, though this approach would introduce complexity to the modeling process.

Another limitation is modeling in-home waste and the inedible portion of foods uses data that is comparatively coarse and dated and may not reflect practices in all countries—especially where nutrients are scarce. For example, fish bones are considered part of the inedible portion, but could be a significant source of calcium in some countries. Canned fish containing fish bones has a very high calcium density score, whereas canned fish with bones removed is low (28).

Only the bioavailability of protein and the IAAs have been included in the analysis as these are largely driven by the protein source itself, rather than other dietary factors. The absorption of calcium, iron, and zinc is impacted by anti-nutritional factors such as phytate and oxalate that are more prevalent in plant-rich diets. This would potentially further reduce the effective supply of these nutrients in some countries. The short-term impact of protein intake and IAA content at the meal level is also outside the scope of this analysis, which assumes all available foods are equally distributed across all meal occasions.

### Impact on protein supply

While much research has emphasized increasing protein supply, our findings indicate that the micronutrients often accompanying protein should receive greater attention. Regarding protein, our results reveal that 570 million people (7.4% of the global population) reside in countries where the protein supply falls short of meeting the adult requirement of 0.8 g of protein per kilogram of body mass per day. Most of these are poorer countries in Sub-Saharan Africa or Latin America. Any steps to increase protein supply must first fit the needs of these people and the supply chains that serve them. This drives toward solutions early in the supply chain, such as improving domestic agricultural productivity.

It is also important to consider the other nutrients that are lacking in these countries to focus on protein sources that are also rich in these nutrients. Using the example of Kenya (Figure 3), in addition to a protein gap there are significant gaps in eleven other nutrients that must also be considered. Selecting the right combination of protein-rich foods may help to address these gaps also, either through a shift in domestic production or trade, noting that some of these gaps exist because the readily traded staple food grains are not good sources of these nutrients.

### Global scenarios for nutrient adequacy

Figure 4 shows the impact of linearly reducing supply targets in countries where there is currently a surplus, on future global requirements for each nutrient. The results shown are based on 2050 convergence to a just adequate global supply of all nutrients.

### Future protein supply

Under the base scenario, global digestible protein requirements decrease through 2050 ending 10% below the total 2020 supply, aligned with the conclusions of Smith et al. (5). For many countries, this represents a significant reduction in protein; for example, the USA would decrease from a sufficiency value of 1.9, China would decrease from 1.75—and given free choice by consumers is unlikely to be realized over the 30 year convergence period, if ever. The sufficiency target is also based on current recommendations of 0.8 g of protein per kg of body weight per day. The 2013 ESPEN expert group (29) suggested a range of 1.0–1.2 g for healthy older adults and 1.2–1.5 g for those malnourished or at risk of malnourishment due to illness.

Using Eq. 2 for target setting and changing the parameters, we can explore a range of different protein supply scenarios for 2050. As noted above converging on a just adequate intake using the current targets, we require 10% less protein in 2050 than was available as food in 2020. If we allow more time to make this transition by pushing the convergence date out to the end of the century, extending the adaptation period by 50 years, this requires a 12% increase in protein by 2050. If the only change is to close the supply gaps where they currently exist, and all other countries maintain their current level of consumption then we require a 26% increase in protein supply by 2050. Adopting an increased 2050 target of 1.2 g/kg/day for everyone, we need an increase of 34%. If everyone on the planet had the protein supply available to China in 2020 (*S* = 1.75) then we require an increase of 57%, and if we converged on the 2020 sufficiency of the USA (*S* = 1.9) then the increase becomes 70%, which is similar to the high-end scenario proposed by Henchion et al. (13).

In all these scenarios, the required increase in bioavailable lysine is smaller than for total protein. For example, increasing the protein target to 1.2 g/kg/day only requires a 12% increase in bioavailable lysine, compared with a 34% increase in digestible protein. This indicates that the increased protein supply could come from lower-quality sources and still meet the required amino acid supply if there was a redistribution of higher-quality protein; that is, many people currently oversupplied could substitute a portion of their animal-sourced protein intakes with plant protein without limiting their protein utilization (as they are likely to be total protein, not IAA limited), making additional animal-sourced protein available to improve the diets of others, or reducing the global need for its production.

### Other nutrients

Looking outside of protein and IAAs, calcium, vitamin A, and riboflavin all showed significant gaps in 2020 supply, requiring increases of 51, 17.8, and 8%, respectively, to close the existing gaps. Using Eq. 2 and projecting forward to 2050, with the added impact of global population growth, we can suggest a range of possible scenarios. Converging on adequate supply for everyone we arrive at an 88% increase in calcium, a 20% increase in vitamin A, and a 13% increase in riboflavin compared to 2020. Closing gaps where they exist now and maintaining current levels of supply where these are above adequate gives calcium +89%, vitamin A +45%, and riboflavin +34%. The comparatively small difference in calcium between these scenarios reflects the small portion of the global population that have a more than adequate food-based calcium supply. If everyone enjoyed the 2020 sufficiency levels of the USA, we get calcium (*S* = 1.18) +121%, vitamin A (*S* = 1.07) +27%, and riboflavin (*S* = 1.75) +95%.

Addressing current and future micronutrient gaps potentially requires much larger food system changes than meeting the basic needs for energy and protein. The scale of change required for many of the micronutrients requires emphasizing foods that are nutrient-dense—have a high level of important nutrients per unit of food energy—to fully nourish people and not just transition from protein-energy malnutrition to hidden hunger and/or obesity.

As previously discussed, converting these targets into realistic food system scenarios requires connecting nutrient requirements back to changes in food production and consumption. This drives toward prioritizing the production of nutrient-dense foods in the most environmentally efficient manner. Beal et al. (22) concluded that countries with adequate energy supply, but inadequate micronutrient intakes should focus on increasing the nutrient density of the foods consumed via a range of different approaches. They found that in most regions of the world, the micronutrient density index has improved over the last 50 years, except for sub-Saharan Africa.

Understanding current levels of inequality provides additional information for scenario models, especially for the near term when the extent of change will necessarily be limited. Changing food consumption patterns globally is a challenging process and is embedded in complex interactions that include prices, preferences, culture, location, and socio-economic status (30), none of which will be resolved rapidly, and potentially, the 30 year convergence period we have used is too optimistic. By setting the length of the adaptation period and a final convergence point for each nutrient, we can set targets for tools like the DELTA Model^®^ that better reflect reality.

## Conclusion

Modeling country-level sufficiency provides valuable insights into the availability of nutrients globally and provides additional perspectives on nutrient undersupply. Many nutrients that appear adequately supplied in global scenarios are undersupplied in many countries, including vitamins A, B12, and B2, and the minerals potassium and iron. Significant increases are required to close some of these gaps.

While the protein supplied in foods globally is already more than sufficient to meet the base needs of the 2050 population if equally distributed, the scale of the inter-country variation means there are significant shortages. A relatively modest production increase of 1%—targeting the needs of countries in deficit—would have closed the 2020 gap. In the absence of any changes in consumption patterns global food protein will need to grow 26% by 2050. A large portion of this growth must be focused on the needs of low-income countries in the form of affordable protein foods that also contain other nutrients that are in short supply, rather than the development of expensive high-tech protein food ingredients.

Any redistribution of nutrients, enabled by reductions in countries currently enjoying an abundant supply, will be a gradual process. Applying a multi-decade linear convergence to country-level sufficiency values provides a useful framework for enabling global food system models such as the DELTA Model^®^ to move from utopian minimum production scenarios toward more realistic assessments of future needs.

While understanding nutrient needs is critical, it is also critical that we translate these into foods produced and diets consumed.

## Data availability statement

The raw data supporting the conclusions of this article will be made available by the authors, without undue reservation.

## Author contributions

AF: Conceptualization, Data curation, Investigation, Methodology, Software, Writing – review & editing. RL: Data curation, Investigation, Visualization, Writing – original draft, Writing – review & editing. WM: Writing – review & editing.
