# Supplementary figures and images for: Analysis of global nutrient gaps and their potential to be closed through redistribution and increased supply
Source: Front Nutr. 2024 Aug 9;11:1396549. doi: 10.3389/fnut.2024.1396549 (PMC11342806; doi:10.3389/fnut.2024.1396549)

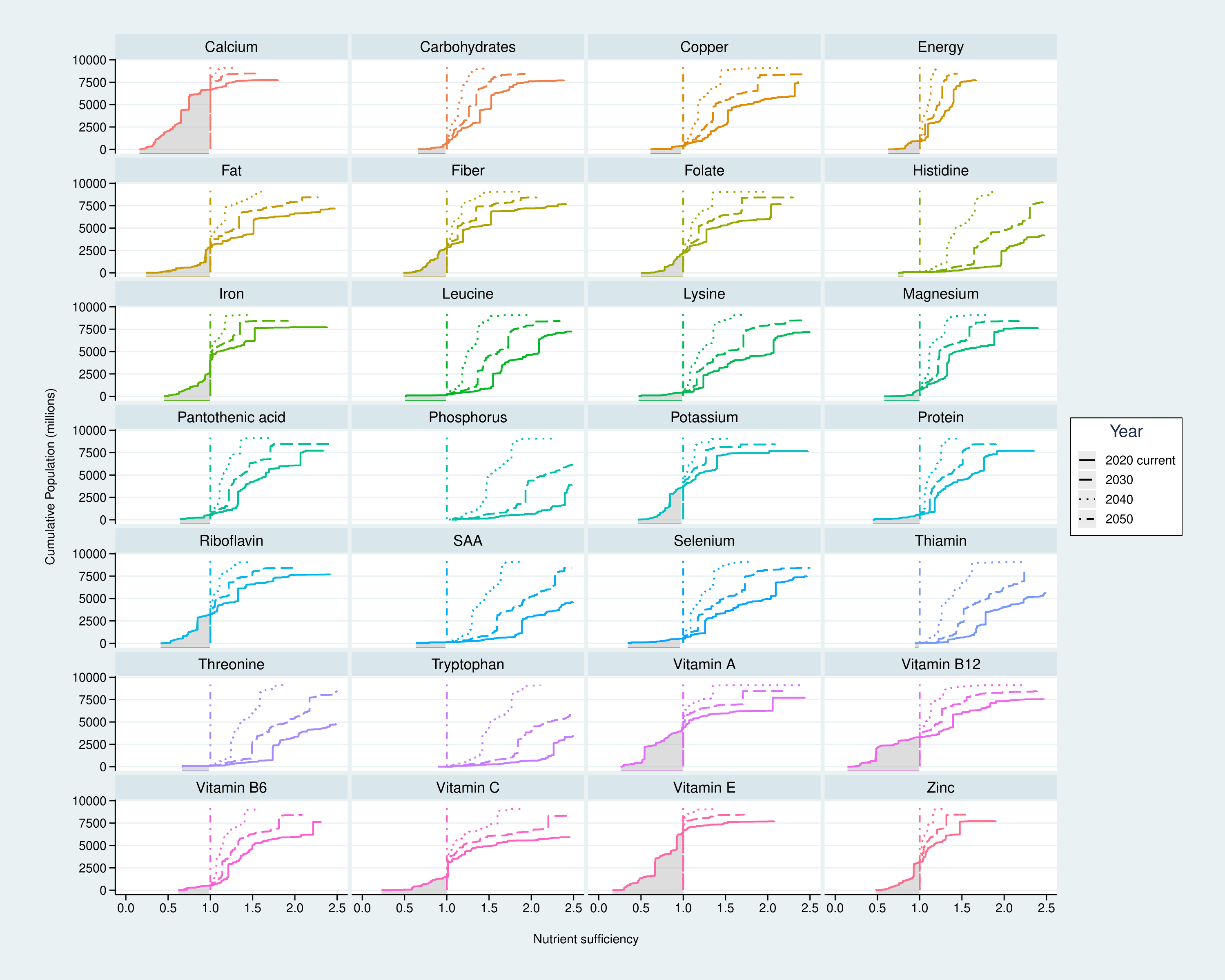

Supplement: Supplementary file 3 [file Image_1.TIFF]
